# Parameters Influence on the Dynamic Properties of Polymer-Matrix Composites Reinforced by Fibres, Particles, and Hybrids

**DOI:** 10.3390/polym14153060

**Published:** 2022-07-28

**Authors:** Zuzana Murčinková, Przemysław Postawa, Jerzy Winczek

**Affiliations:** 1 Department of Design and Monitoring of Technical Systems, Faculty of Manufacturing Technologies with Seat in Prešov, Technical University of Košice, Bayerova 1, 080 01 Prešov, Slovakia; 2 Department of Technology and Automation, Częstochowa University of Technology, Al. Armii Krajovej 19C, 42-201 Częstochowa, Poland; przemyslaw.postawa@pcz.pl (P.P.); jerzy.winczek@pcz.pl (J.W.)

**Keywords:** modulus, loss factor, logarithmic decrement, fibre material, fabric weaving, fibre orientation, temperature, frequency, particle size, polymer matrix

## Abstract

In this paper, we present an extensive experimental study on the dynamic mechanical properties of composites with polymer matrices, as well as a quantification of the parameters that influence these properties. Polymer-composite matrices make it possible to form any reinforcement arrangement of fibres, particles, and layers, which makes it possible to form composite materials with certain dominant mechanical properties according to the internal arrangement for the application. In this study, we focused on the dynamic properties (i.e., damping parameters, such as the loss factor (tan *d*), logarithmic decrement (*λ*), storage modulus (*E*′), and loss modulus (*E*″)) of composites with polymer matrices, including parameters such as the fibre material, fabric weaving, fibre orientation, temperature, frequency, particle size, volume of short fibres, and epoxy resin type. If other articles focus on one type of composite and 1–2 parameters, then the benefit of this article lies in our analysis of 8 mentioned parameters in the experimental analysis of 27 different types of composites with polymer matrices. The tested fibre materials were glass, aramid, and carbon; the tested woven fabrics were twill, plain, unidirectional, and satin; the temperature range was from −50 to +230 °C; the frequency was 1 Hz and 10 Hz; the particle size was 0.1–16 mm; the volume percentages of the short fibres were 3, 6, and 12 vol.% of the hybrid polymer composites and the type of polymer matrix. We used the free-damped-vibration method with vibration dynamic signal analysis and the forced-damped vibration of dynamic mechanical thermal analysis for testing. We ranked the parameters that influence the dynamic vibration properties according to the effects. Among sets of results provided in the paper, considering the storage modulus, loss modulus, and loss factor, the best results of the fibre composites were for aramid-fibre-reinforced polymers, regardless of the weave type, with an advantage for unidirectional fabric. The best results of the particle composites were for those with fine filler sizes that incorporated the short fibres.

## 1. Introduction

The source of the macroscopic mechanical behaviour of a material is its internal arrangement. For multilayered laminates, we determine the internal arrangement by reinforcement fabric patterns woven with uni-, bi-, and multidirectional weaving fibres, as well as the particular dimensional characteristics of the fibres and layup of the laminate. For particular composites, the internal architecture can be variable mainly for reinforcement dimensions and shapes.

In the classical approach, the materials are supposed to be homogeneous; however, each material has its own meso-, micro-, or nanostructure. The material homogeneity represents the idealisation of the material continuum. For example, in steel, we distinguish the components ferrite, martensite, perlite, and austenite, which may also include alloys. We can arrange the components differently, with different grain sizes and different mechanical microproperties, which fundamentally affect the macroscopic mechanical properties and behaviour of steel, which is the same for other anorganic materials and biological tissues (wood, bones), which are all of a hierarchical structure with the internal arrangement, configuration or architecture of the material [1,2,3].

In the current publications, researchers focus mainly on the strength and stiffness of composite materials, the method of the analytical prediction of these quantities, and multilevel simulations and experiments for individual types of composites. In the numerical and experimental studies, researchers not only analyse the static properties, but also the impact or combined ones [4,5,6,7,8,9]. However, in industrial applications, the composites are not only subjected to static loads, but also to the dynamic components of the loads, which are substantial, along with vibrations as natural parts of the mechanical-system operation. The utilisation of vibration dynamic characteristics [6,10] for applications is an important scope of the current research. Computations of composite materials and hybrid-structure materials require advanced models and computation (i.e., multilevel models, representative volume element models, micromechanical models, etc.) that use finite element analysis, boundary element methods, or meshless methods that focus on linear and nonlinear static and dynamic analyses and optimisation. The numerical simulations represent a demanding and complex process of modelling complex composite structures of multilayer laminates, as well as particle composites [11,12,13,14].

Recently, researchers have investigated the dynamic mechanical properties, as well as their utilisation and control. Researchers tested high-damping sandwiches, and they found that the mechanical and damping properties deeply depend on the fibre orientation of the lamina, the property of the rubber sheet (involved in the sandwich laminate), and its layer number and sequence [15]. In the development of the field of polymer composites, researchers have focused on testing different volume fractions of individual composite constituent phases, filler sizes, and their optimum compositions (more in [16,17,18]). Researchers found that polymer composites (both layered and particulate) had a significant effect on the damping for applications of high-frequency vibrations, and they measured a reduction in the resonant peak up to 30%, mainly by using polymer matrices (more in [19,20]). Researchers have investigated the field of applications that use polymer concrete for a machine base frame or to fill existing machine designs to improve the dynamic stiffness in advanced applications of production-machine components for precision-tool machines [16,21], a grinding machine [22], and a machine-tool worktable [23]. Polymer-matrix composites can be both machined [24] and a component part of machine-tool frames. The vibration dynamic characteristics affect the noise emissions [25], which is an important factor in traffic and production factories that influences everyday life. 

In the last years, carbon nanofibers (CNFs) have shown wide applications in the fields of materials science, nanotechnology, energy storage, environmental science, biomedicine due to their unique structures and functions, and a lot of achievements on the synthesis and application of CNF-based nanomaterials have been obtained [26]. CNFs as reinforcing phase of composites can form various CNFs with porous, stacked-up, helical, and tubular structures [26] and, thus, allow for the creation of even more complex internal material structure. Moreover, due to the rich intertube contacts and various ways to dissipate energy, carbon nano-tubes assemblies have shown great advantages for developing novel high-damping materials, which can also show high strength or modulus for engineering applications [27].

A general comprehensive view of the parameters that influence the dynamic properties, and a quantification of the contributions of the individual parameters, are lacking in the previous work. We can state that, in general, for fibre and particle composites, the internal structure and arrangement contribute to the influence and control over the mechanical properties. Moreover, frequency and temperature can influence the dynamic mechanical properties of materials. The contribution that we make in this study is the focus on the determination and ranking of the highest and lowest effects and contributions of each of the following parameters to the dynamic mechanical properties (i.e., the storage modulus (*E*′), loss modulus (*E*″), and damping measures as the loss factor (tan *d*) and logarithmic decrement (*λ*)):

- For the fibre composites, the effects of the fibre material, fabric weaving, fibre orientation, temperature (large range: −70 to 200 °C), and frequency (1 Hz and 10 Hz);
- For the particle composites, the effects of the particle size, short-fibre-volume percentage, and type of polymer matrix.

Furthermore, we used two experimental methods for the estimation of the mentioned dynamic properties, depending on the composite type: dynamic mechanical thermal analysis and vibrodiagnostics. Researchers use dynamic mechanical analysis as a general evaluation method to research the viscoelastic behaviour of polymers. Vibrodiagnostic methods allow for the monitoring and analysis of the dynamic signal to research the vibration-damping characteristics.

## 2. Materials and Methods

According to the shapes and dimensions of the samples of the two types of composite materials, we chose two experimental measurement methods, which we describe in the following sections, to determine the dynamic properties.

### 2.1. Dynamic Mechanical Thermal Analysis: Forced Vibration Test

**Figure 1:**
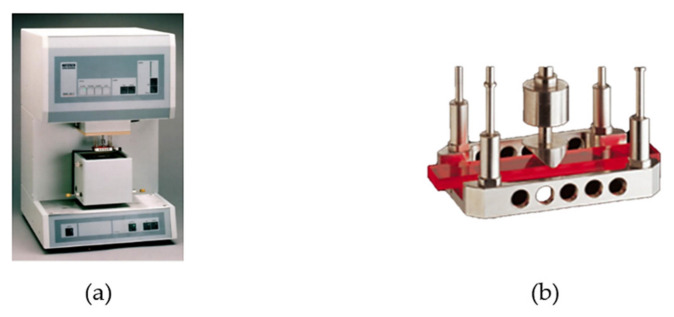
(**a**) Dynamic Mechanical Analyser DMA 242C device by Netzsch Selb, Germany, and (**b**) view of sample holder for 3 point bending tests.

Dynamic mechanical thermal analysis (DMTA) is one of the thermal analysis methods that is designated specifically for polymers because of the substantial impact of temperature on mechanical properties. For most polymer materials and their composites, the glass transition temperature is important because of the change in the structure and stiffness. The DMTA test is based on forced oscillations and the measurement of the force change during the three-point-bending deflection of the sample, which, in the present study, we assessed over a wide range of temperatures. The equipment provides the possibility of different states of deformation (free bending, dual cantilever, tension, and compression). Sensors read the tested material response during the deformation of the sample and measure the force and time of the response between the dynamic force of the pushrod and the sample. We present the schema of the measurement elements of DMTA and the sample holder that we used in the experiment in Figure 1.

**Figure 2:**
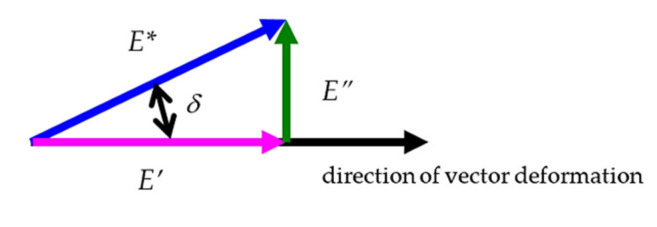
Schema of relations among quantities.

The major parameters that define the viscoelastic nature of polymer composites are the storage modulus (*E*′), loss modulus (*E*″), phase angle (*δ*), and loss factor (tan *δ*) (notated as tan *d* in the rest of the paper), which we can obtain during DMTA investigations. We schematically show the relations among the mentioned quantities in Figure 2, which are as follows:

(1) $$E^{\prime \prime }=E^{\prime }\tan\delta$$

and

(2) $$E*=E^{\prime }+iE^{\prime \prime }$$

### 2.2. Vibration Diagnostics and Bump Test: Free-Vibration Test

We performed the bump test with dynamic vibrational signal analysis at room temperature, as the shapes of Samples A–I and the capabilities of the DMTA analyser did not allow us to determine their damping parameters.

The tested material sample represents the SDOF (single degree of freedom) mass–damper–spring vibration system. The bump force excites the damped-free-vibration response (more in [28,29]). The samples freely vibrate on a natural frequency(ies) and are damped by the internal friction of the material. The equation of the underdamped motion (damping ratio: *ζ* < 1) for the free-damped vibrations of an SDOF mechanical system is:

(3) $$x(t)=X_{0\;}e^{-\zeta \omega_{n}t}\sin\left(\omega_{d}t+\varphi \right)$$

where *x* is the underdamped displacement (amplitude, distance from equilibrium) of the mass for the free-vibration effect of the mechanical system, *X*_0_ is the amplitude, *ω*_n_ and *ω*_d_ are the undamped and damped natural frequencies, respectively, and *φ* is the phase angle.

According to (3), we can write the amplitudes of two successive vibration cycles (*x*_1_ and *x*_2_) as:

(4) $$\begin{array}{l} x_{1}(t_{1})=X_{0\;}e^{-\zeta \omega_{n}\;t_{1}}\sin\left(\omega_{d}t_{1}+\varphi \right)\\ x_{2}(t_{2})=X_{0\;}e^{-\zeta \omega_{n}\;\left(t_{1}+T_{d}\right)}\sin\left(\omega_{d}\left(t_{1}+T_{d}\right)+\varphi \right) \end{array}$$

where $t_{2}=t_{1}+T_{d}$, and *T*_d_ is the damping period.

We can define the logarithmic decrement (*λ*), which represents the rate at which the amplitude of a free damped vibration decreases, over a number of cycles (*n*):

(5) $$\lambda =\frac{1}{n}\ln\frac{x_{i}}{x_{i+n}}$$

where *x_i_* and *x_i+n_* are the amplitudes of vibration cycles *i* and *i + n*.

**Figure 3:**
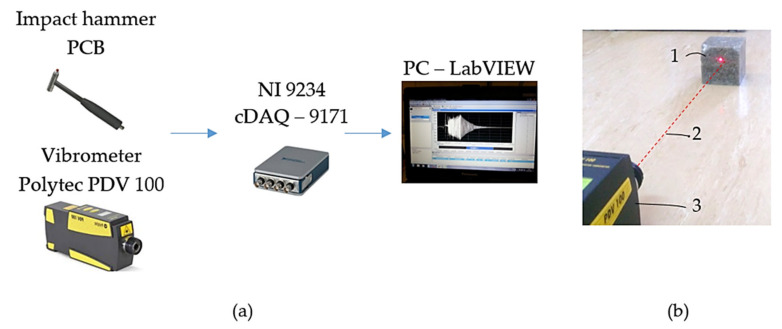
(**a**) Measurement chain, (**b**) details of measurement (1: sample; 2: laser beam; 3: vibrometer).

The measurement setup involves the following equipment (Figure 3a):

- Portable Digital Vibrometer (PDV 100), for noncontact vibrational measurement in a frequency range from 0.5 Hz to 22 kHz, and at a variable working distance from 0.1 m to 30 m, and an operating temperature from +5 °C to +40 °C (Polytec GmBH, Waldbronn, Germany);
- NI 9234 Sound and Vibration Input Module, with a dynamic range of 102 dB. The input channels sample at rates as fast as 51.2 kS/s, with a 24-bit resolution, 50 g of shock, and 5 g of vibration (National Instruments Corporation, Austin, TX, USA);
- CompactDAQ Chassis cDAQ-9171 (National Instruments Corporation, Austin, TX, USA);
- An impact hammer (PCB) to mechanically impact the samples by bump force, with a sensitivity of 2.25 mV/N, and a measurement range of ±2224 N pk (PCB Piezotronics, Depew, NY, USA);
- Software for an advanced analysis of the dynamic signal based on LabView Sound and Vibration Toolkit software (National Instruments Corporation, Austin, TX, USA).

In Figure 3b, we present the sample and vibrometer during measurement. The red dot is the contact point of the laser beam (red line). The time-domain-response functions of the sample were the output signal. To estimate the values of the logarithmic decrements, we made two time records, i.e., each time record for one bump excitation. The average values are provided in the results.

### 2.3. Samples and Materials

The testing samples were polymer-matrix-composite materials. In the following section, we provide a description of the fibre and particle composite samples that we used in the experimental study. We incorporated continuous glass, aramids, and carbon into the polymer matrix in the form of fabrics in Samples 1–12, as they are the most common fibre reinforcements. Samples A–I consisted of spherical particles (preferably silica) and spherical particles with discontinuous fibres (carbon), which we distributed in the polymer matrix. Individual samples differ in the various materials, shapes, sizes, arrangements, orientations, volume percentages of reinforcement, etc.

#### 2.3.1. Multilayered Laminate Composites

The tested laminates were glass-fibre-reinforced polymers (GFRPs), aramid-fibre-reinforced polymers (AFRPs), and carbon-fibre-reinforced polymers (CFRPs). Fibres were continuous in the form of woven bidirectional and unidirectional fabrics.

Samples 1–12 (Figure 1) consisted of six layers of fabrics in the polymer matrix. Each layer had the same orientation, characterised by the warp fibres situated in the longitudinal dimension of the samples and corresponded to 0°. The dimensions of samples were 10 × 50 mm, with a thickness range of 0.2–1.6 mm. The polymer matrix was epoxy resin (LG285) with an HG285 curing agent at a mixing ratio of 100:40. Resin LG285 is a high-tech laminate designed for hand lamination without heat postcuring. The mentioned resin is mainly used in the aviation industry. The producer in [5,30] describes the mechanical properties of the epoxy resin (LG 285) with HG286.

**Figure 4:**
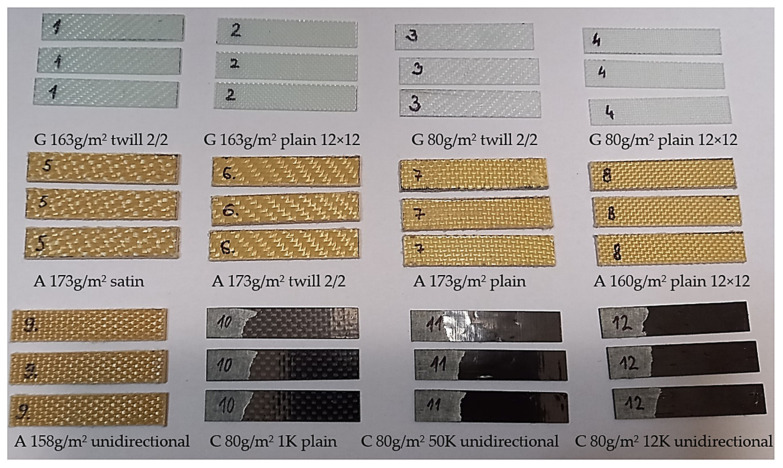
Samples 1–12, multilayered laminate composites, samples: 1–4 with glass fibre fabrics (G), 5–9 with aramid fibre fabrics (A), and 10–12 with carbon fibre fabrics (C) of mass per unit area 80–173 g/m^2^ and twill, plain, satin, and unidirectional weave; 1 K, 50 K, and 12 K is yarn size warp; 2/2 is 2-over, 2-under plain weave pattern; 12 × 12 is yarn density warp x weft.

In Figure 4, we present three of each type of sample that were tested. Moreover, we provide the basic characteristics of the 2D fabrics in the figure (i.e., letters G, A, and Care for glass, aramid, and carbon, respectively). The mass per unit area of fabrics was in the range of 80–173 g/m^2^, and the types of woven fabrics were twill, plain, unidirectional, and satin.

**Figure 5:**
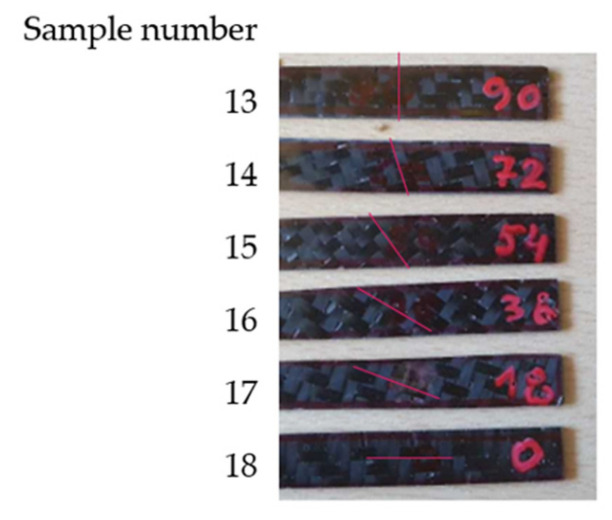
Samples 13–18 with orientations of warp fibres: 0°, 18°, 36°, 54°, 72°, and 90°.

We present the sets of bidirectional three-layered carbon twill-weave-fabric (200 g/m^2^) samples in Figure 5. The six different orientations (i.e., 0°, 18°, 36°, 54°, 72°, and 90°) of the warp fibres are for Samples 13–18. Three layers of each sample were of the same warp-fibre orientation. Dimensions were 10 × 50 × 1 mm. Epoxy resin was EPIKOTE TM Resin MGS LR 285 and curing agent was EPIKURE TM MGS LH286, at a mixing ratio of 100:40 (for technical information, see [31]).

#### 2.3.2. Particle-Reinforced Composites

We uniformly distributed the particle reinforcement (filler) with a random orientation, and of a spherical shape. The particles were in the forms of silica gravel, sand, and dust of various fractions in the range of 0.1–16 mm. We can recognise them as spherical; however, the particle shapes are not ideal spheres and, even at the macroscopic level, we could identify roundness, sphericity, and regularity. Samples A–I are of a cube shape, with dimensions of 100 × 100 × 100 mm.

**Figure 6:**
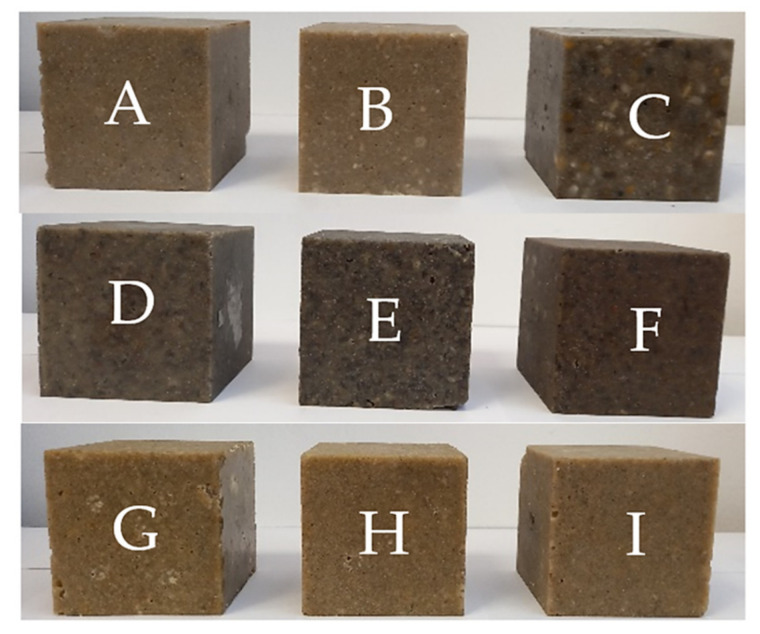
Samples (**A**–**I**), particle-reinforced composites, samples: (**A**–**C**) with different filler sizes (more in Table 1), (**D**–**F**) with different short fibre volume and same fillers (more in Table 2), and (**G**–**I**) with different epoxy resins and constant fillers mixture (more in Table 3).

We made the first set, Samples A–C (Table 1), to test the dimensions of the filler dependent on dynamic quantity (i.e., damping measured by logarithmic decrement). In the second set, Samples D–F (Table 2), we focused on the influence of the volume percentage of chopped short carbon fibres in the range of 3–12 vol% on the damping, and we used the last sample set, Samples G–I (Table 3), to test the influence of various resins and fixatives on the damping. We present the samples in Figure 6.

## 3. Results and Discussion

### 3.1. Results of DMTA Tests of Multilayered Laminates

**Figure 7:**
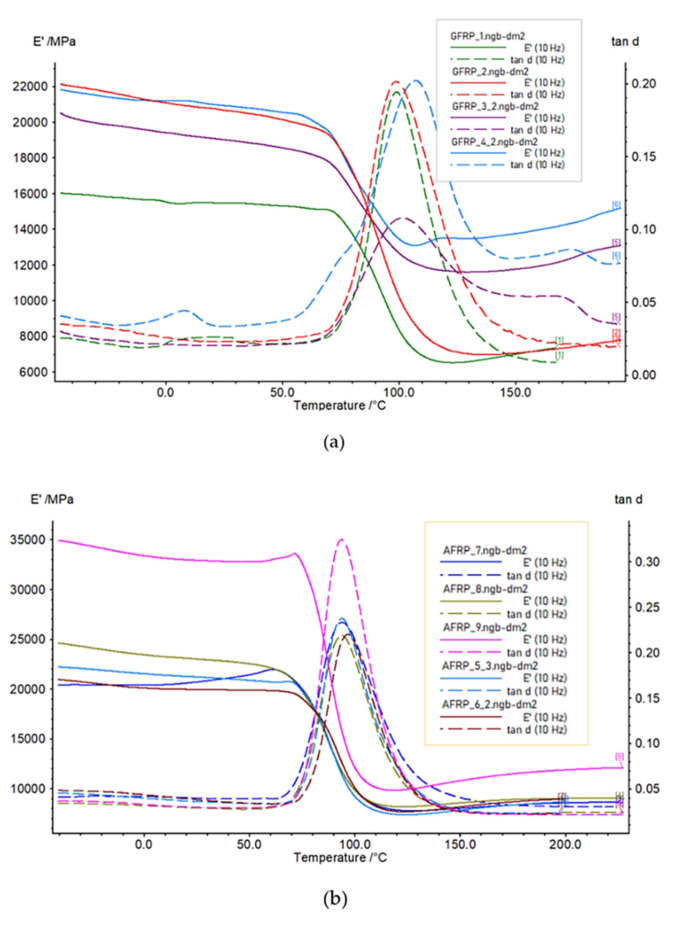

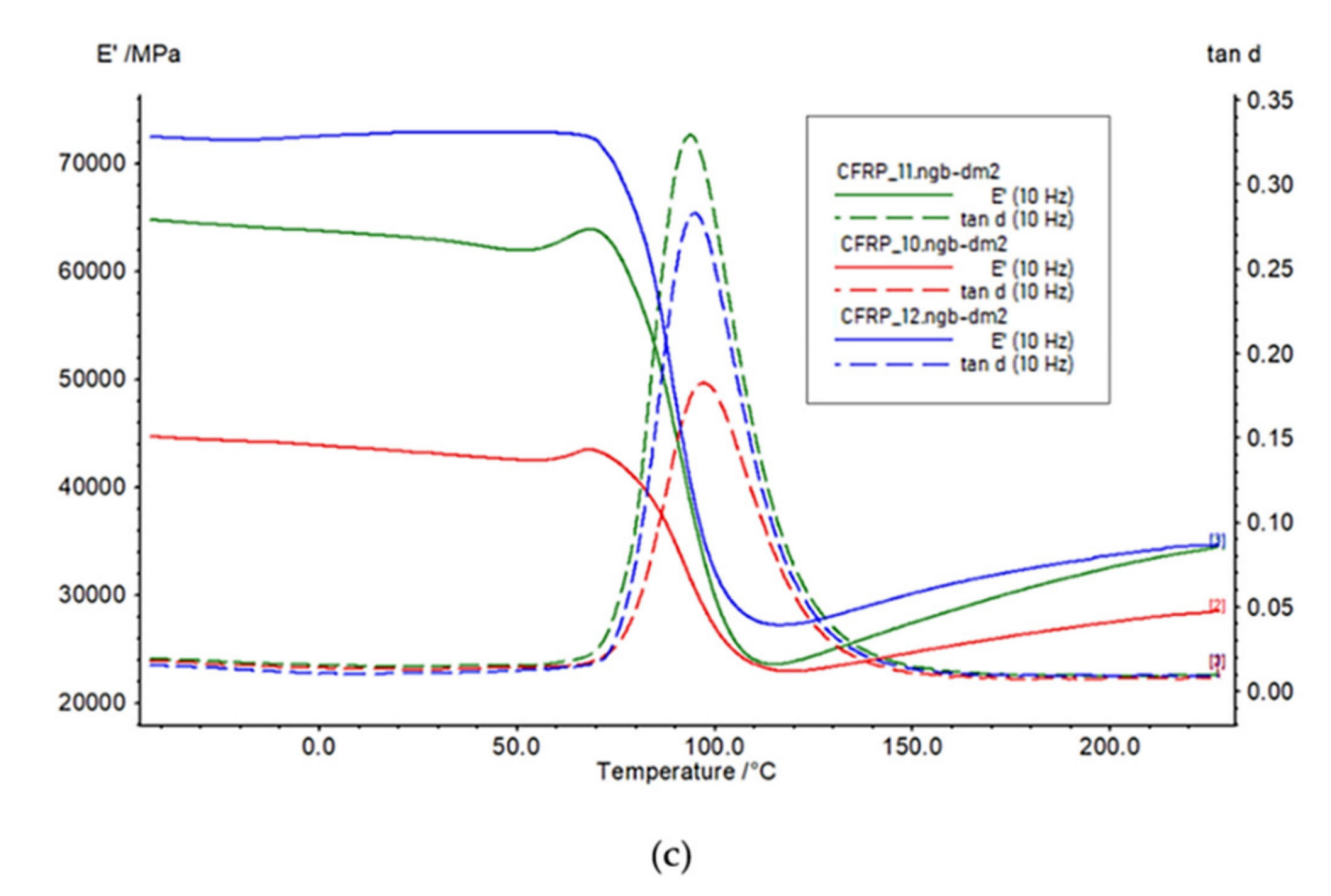
DMTA results of: (**a**) GFRP, (**b**) AFRP, and (**c**) CFRP. The samples were subjected to a frequency load of 10 Hz.

The presented results are for the samples that we describe in more detail in Section 2.3.1. We analysed the variations in the storage and loss modules and the loss factor along the temperature at a constant frequency. The temperature while testing was in ranges from −50 to 200 °C and from −50 to 230 °C, depending on the thermal conditions, with a heating rate of 2 K/min. The results that we provide in Figure 7 are for a dynamic load force of 6 N, a frequency of 10 Hz, and an oscillation amplitude of 120 µm.

The nature of the fibre reinforced polymer plots in Figure 7 is typical for native polymers without reinforcement. It indicates very strong influence of the polymer matrix on the mechanical nature of the polymer matrix composite behaviour. However, the individual laminate composites show different values for the loss factor and storage modulus, which significantly depend on temperature. In the range of the standard operation conditions (i.e., 20–50 °C), the storage modulus is that which differs the most when comparing the individual samples. At a temperature of about 70 °C, the storage modulus begins to decrease and the loss factor increases. The unidirectionally oriented fibres improved the stiffness the most, as well as the damping capacity in the glass transition (Tg) temperature.

**Figure 8:**
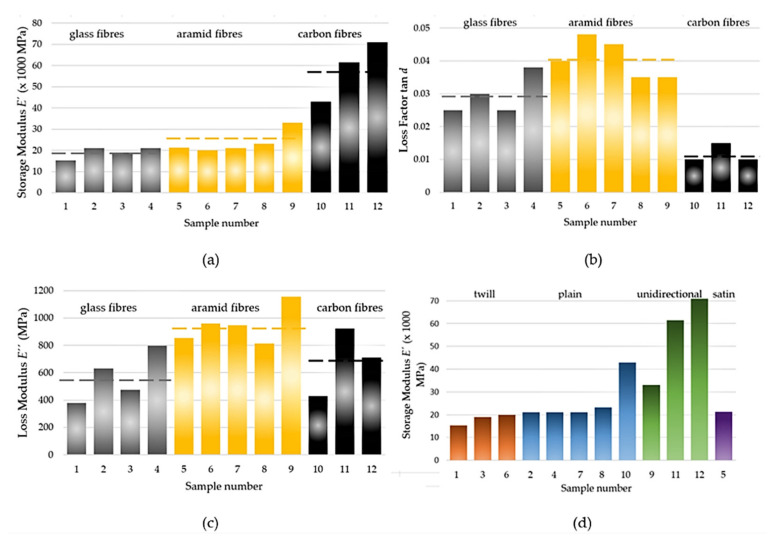
Effects of fibre material on: (**a**) storage modulus (*E*′), (**b**) loss factor (tan *d*), (**c**) loss modulus (*E*″), and (**d**) the effect of weaving on *E*′; dashed lines represent the average values.

We processed the results in Figure 8 as the average values in the temperature range from −50 to 60 °C for the storage modulus (*E*′), loss factor (tan *d*), and loss modulus (*E*″), which we compared. By making a comparison according to the material of the fibre (Figure 8a), regardless of the weave type and weight per m^2^, the GFRP samples had the lowest values of the storage modulus (*E*′, and the AFRP and CFRP samples had average values in the mentioned temperature range that were 1.35-times and 3.07-times higher, respectively. The ability to store energy elastically (i.e., the ratio of the elastic (in phase) stress to strain) was the largest for the CFRP.

Another parameter for comparison is the loss factor (tan *d*), which indicates the damping of the material (i.e., degree of energy dissipation) (Figure 8b). The lowest loss factor was for the CFRP, and the GFRP and AFRP samples had 2.52-times and 3.48-times larger loss factors in the range of −50 to 60 °C, respectively.

Moreover, we can evaluate the loss modulus (*E*″) (Figure 8c) according to (1). The loss modulus reflects the energy-dissipation capacity and the material’s ability to dissipate stress through heat. This means that the ratio of the viscous (out of phase) component to stress was the largest for the AFRP. The GFRP and CFRP samples had average values that were 0.6-times and 0.73-times lower in the evaluated temperature range from −50 to 60 °C.

The influence of the weaving type (Figure 8d), regardless of the material, was obvious after evaluating the storage modulus (*E*′). The orientations of the warp fibres were the same in all the layers, and so the orientation towards the direction of the loading force was the same. The unidirectional-fibre-orientation samples (i.e., 9, 11, and 12) had the highest values. The twill, plain, and satin weavings had similar values, which were slightly better for the plain weave.

CFRPs are characterised by a special relationship among the measured quantities. CFRPs have a low loss factor, even though the loss modulus (*E*″), which represents the ability to dissipate energy, is high and comparable to AFRPs. In our case, the large storage moduli of the CFRPs of Samples 11 and 12 were amplified because of the unidirectional fibres. The source of the low loss factor of the CFRP is its large storage modulus (*E*′), as a measure of the material stiffness and the ability to store energy during one load cycle. Thus, the carbon and aramid fibres are a good combination for hybrid carbon–aramid-fibre-reinforced composites (more in [32]), which are characterised by both high storage and high loss moduli, as well as a high loss factor.

Bidirectional and multidirectional fabrics are intended for the multiaxial stress that is applied for structural components. However, the composites of unidirectionally oriented fibres are characterised by fibre-direction-dominant properties that are larger than those of bi- and multidirectional fabrics. The unidirectional woven fabric, according to the unidirectional (dominant) stress in the final component subjected to a unidirectional load, allows us to maximise its mechanical properties.

**Figure 9:**
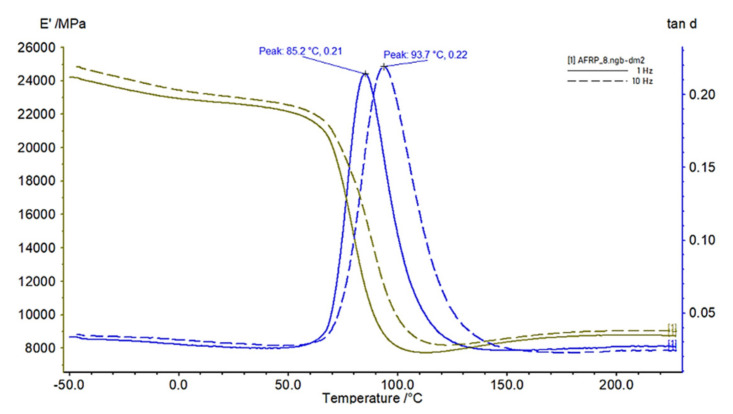
Effects of frequencies (Sample 8, AFRP): 1 Hz (continuous line); 10 Hz (dashed line).

The dynamic properties of materials depend on the frequency of excitation force (Figure 9). After we evaluated the loss factor (tan *d*) at the glass transition (Tg) temperature for 1 Hz and 10 Hz for Samples 1–12, the increase in the loss factor (tan *d*) was in the range of 0–22.2% (Table 4).

**Figure 10:**
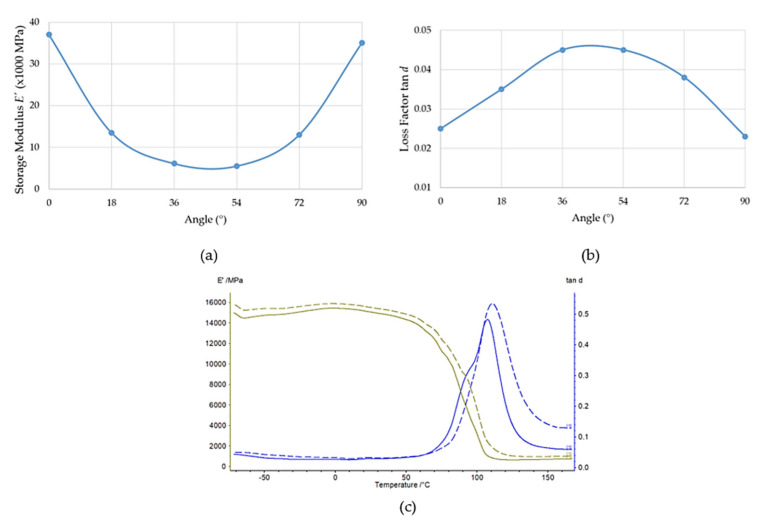
Effects of (**a**) *E*′ and (**b**) tan *d* on angle of warp fibres of Samples 13–18 for 60 °C and a frequency of loading force of 1 Hz, and (**c**) DMTA plot of Sample 14 (bidirectional fabric, 18°).

Each woven fabric had warp and weft fibres that we could orient differently with respect to the load. We present the significant effect of this parameter in Figure 10a,b. The nature of the curves in Figure 10a,b is the same as for those samples in the whole range of −50 °C to 60 °C. The storage modulus (*E*′) is minimal for 45° orientation and loss modulus (tan *d*) is the maximal for that orientation. In our case, difference between storage modulus *E*′ and loss factor tan *d* of 0° (sample 13) and 54° (sample 16) is significant, i.e., by 85%. We present the DMTA plots of the storage modulus (*E*′) (green) and the loss factor (tan *d*) (blue) of Sample 14 in the range from −70 °C to 170 °C and for frequencies of 1 Hz (continuous) and 10 Hz (dashed) in Figure 10c as representatives of all the plots made for evaluation.

### 3.2. Results of Bump Tests of Particle-Reinforced Composites

**Figure 11:**
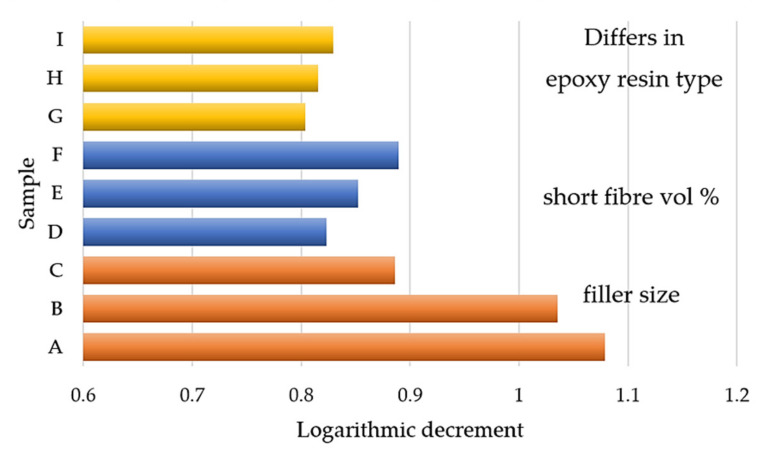
Logarithmic decrements of particle composites: Samples A–I.

Samples A–I differ in the filler size and shape (Figure 11). The smallest fractions of filler for A and B provided the better logarithmic decrements. In terms of damping, smaller fractions are more suitable. Thus, the larger filler–matrix contact area (i.e., interfacial surface) and the larger number of fillers substantially contributed to the larger dissipation of the vibration energy. Sample C had the largest fraction. However, its damping was larger than those of the others. The positive source of the damping in Sample C was silica sand (0.3–1 mm), which had 30 vol% of filler (Table 1).

We incorporated chopped carbon fibres of 3, 6, and 12 vol% of the medium fraction filler into Samples D–E (more in Table 2). The higher volume percentage of the chopped fibres improved the damping. By comparing a 3 vol% and 12 vol%, the damping was increased by 8%.

We used Samples G–I to compare the logarithmic decrements of the three different epoxy resin matrixes with the same filler (Table 3), but with different rates of solidification during processing. The maximal difference is 3%. The type of epoxy resin matrix is the least influential parameter for damping.

### 3.3. The Nature of Time-Domain-Response Curves

**Figure 12:**
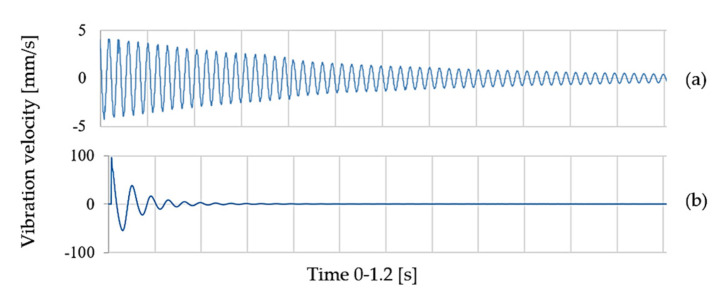
Time-domain-response curves: (**a**) multilayered laminates, and (**b**) particle composites.

The time-domain responses of the multilayered laminates and particle composites of same boundary conditions were substantially different in terms of the number of vibration cycles and amplitudes. We compared the curves in Figure 12 over the same time (i.e., 0–1.2 s). In the case of multilayered laminate samples (Figure 12a), the response curve is characterised by the number of cycles in the mentioned time period. The number of cycles (*n*) is in the order of tens to hundreds. However, we can see from the character of the time-domain curves of the particle samples in Figure 12b that only three–six cycles are necessary to damp the bump force, although the first amplitude is 20 times larger. The time required to damp the free vibrations is very short. Such a composite material is characterised by a substantial damping time and measures, which we cannot achieve with the traditional construction materials.

### 3.4. Microfield Distribution and Sources of the Composite Material Damping

**Figure 13:**
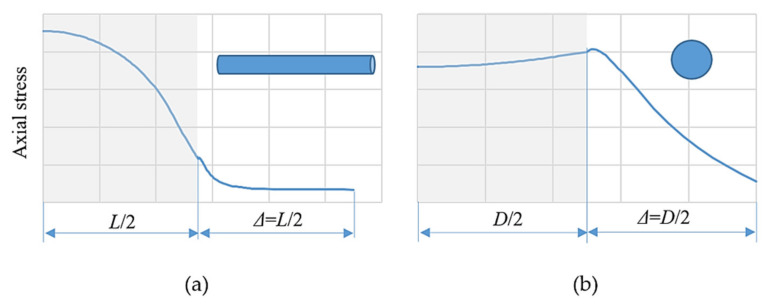
Axial stress in half of (**a**) fibre/(**b**) particle: *L*/2 is half of fibre length, *D*/2 is half of particle diameter, *Δ* is gap between fibres/particles.

The mechanism of composite materials damping is still object of research. To understand the source of the vibration-energy dissipation and obtain the mechanical microfield distribution, we analysed the unit cells in static numerical analyses by using the finite element method. The numerically simulated unit cells consisted of a single fibre/particle surrounded by a matrix. We obtained the axial-stress distributions for the short cylindrical fibre and spherical particle by using the symmetry boundary conditions and prescribed displacement in the fibre/particle axis. The ratio of the Young’s modulus of the matrix and fibre (*E*_m_/*E*_f_) was 1:100. The aspect ratio between the fibre diameter and its length (*D*/*L*) was 10. The particle was of a spherical diameter (*D*). Regardless of the volume fraction of the fibre/particle, the character of the axial-stress distribution was the similar. (Figure 13, grey section is axial-stress distribution in half of fibre/particle, distribution is symmetrical in the second half).

Comparing the characters of the axial-stress distribution in the fibre and in the particle axes, they are different. For the spherical-particle composites, the gradient of the axial stress, as well as its maximum value, is the largest in the matrix in the vicinity of the particle/matrix interface. Applying the dynamic periodic force, the maximum of that stress is damped by the material of the matrix itself which is one of the sources of damping. In contrast, for short-fibre-reinforced composites, the maximal axial stress is in the fibre which has lower material damping as s matrix. However, a source of damping is the maximum shear stress at the surface of fibre ends and it is the initiator of slip caused by decohesion and recohesion that increases the damping capacity. During the slip process, energy is dissipated through heat caused by the frictional sliding at the interface [33].

The fibre/matrix interfaces are subjected to elastic as well as plastic deformation under vibration conditions [34]. We recognize the frictional damping mainly due to slip in the unbound regions between the fibre/matrix interface as damping due to the damage [35]. The fibre/matrix interface is a stress-transfer medium, and it determines the composite performances in failures that are initiated by the accumulation of interfacial cracks [36].

## 4. Conclusions

The polymer matrix secured the position of the reinforcement or filler; however, the nature of that material directly contributed to the dynamic properties of the tested composites. In this paper, we offer the results of a broad experimental analysis of the polymer matrix based on free- and forced-damped vibration tests, in which we mainly focused on factors that affect the dynamic properties, such as the fibre material, fabric weave, fibre orientation, temperature, frequency, particle size, short-fibre-volume percentage, and type of epoxy resin matrix. We tested 27 samples of composites. We highlight the following conclusions from the experimental study:

○ For the multilayered laminates, the fibre orientation had the greatest effect, followed by the fibre material;
○ The fibre orientation had a greater effect than the type of weaving;
○ The type of weaving affected the storage modulus (*E*′) more than the tan *d*;
○ In the range of temperatures from −50 to 60 °C, the loss factor (tan *d*) was about 10-times lower than at the Tg temperature;
○ The loss factor (tan *d*) at Tg temperature was in the range of 0–22.2% when we compared loading frequencies of 1 and 10 Hz of multilayered laminate composites samples;
○ The particle size had the greatest effect on the particle composites damping.

The time-domain responses of the multilayered laminate and particular composites were very different and were shown in the number of cycles and amplitudes, as well as in other resulting quantities, such as the damping time. For particular composites, the number of vibration cycles was from one to two orders lower.

Considering the storage modulus (*E*′), loss modulus (*E*″), and loss factor (tan *d*), the best results among the 12 presented fibre-reinforced laminate-composite samples are for multilayered AFRPs, regardless of the fabric weave type, with an advantage for unidirectional fabric. The advantage of increasing the stiffness is the involvement of carbon. The largest loss factor of six CFRPs of bidirectional fabrics was for a 45° orientation of the warp fibres towards the direction of the acting load. The best results of nine particle and hybrid composites were for those with fine filler sizes that incorporated the short fibres.

In applications of dynamic loaded composite components, the material damping is a factor that is significant compared to steel as standard material in mechanical engineering. Thus, the passive damping of composite materials is an important and notable aspect of designing and the designers can control and maximize it by influencing the parameters evaluated in the presented paper.

## Figures and Tables

**Table 1:** Descriptions of Samples A–C with different filler sizes.

	Filler:Matrix (Vol%)	Fillers Vol%	Filler Fraction
**A**	75:25	50	Silica sand (**0.3–1 mm**)
		50	Silica dust (**0.1–0.3 mm**)
		100:16	epoxy resin CHS-EPOXY 324:fixative TELATIT 0492
**B**	75:25	50	Silica gravel (**2–4 mm**)
		50	Silica dust (**0.1–0.3 mm**)
		100:16	epoxy resin CHS-EPOXY 324:fixative TELATIT 0492
**C**	84:16	35	Danube gravel (**8–16 mm**)
		35	Danube gravel (**4–8 mm**)
		30	Silica sand (**0.3–1 mm**)
		100:50	epoxy resin LH 160:fixative H 287

**Table 2:** Descriptions of Samples D–F with different short fibre volume and same fillers.

Filler:Matrix Ratio: 75:25 Vol% Epoxy Resin LH 160:fixative H 287: 100:50	D	E	F
Chopped carbon fibres (3 mm) (vol%)	**3**	**6**	**12**
Andesite gravel (4–8 mm) (vol%)	49	48	46
Silica sand (0.3–1 mm) (vol%)	24	23	21
Silica dust (0.1–0.3 mm) (vol%)	24	23	21

**Table 3:** Descriptions of Samples G–I with different epoxy resins and constant fillers mixture.

Filler:Matrix 78:22 Vol%		
50%		Silica gravel (2–4 mm)
30%		Silica sand (0.3–1 mm)
20%		Silica dust (0.1–0.3 mm)
**G**	Ratio of epoxy resin **LH 160**:fixative **H 287**: 100:50	
**H**	Ratio of epoxy resin **LH 289**:fixative **H 287**: 100:50	
**I**	Ratio of epoxy resin **CHS-EPOXY 517**:fixative **P11**: 100:11	

**Table 4:** Loss factor at Tg temperature.

Sample	1	2	3	4	5	6	7	8	9	10	11	12
**Frequency**	1 Hz											
**tan *d* (-)**	0.19	0.19	0.09	0.18	0.23	0.21	0.23	0.21	0.32	0.18	0.31	0.28
**Tg (°C)**	89.8	89.5	91.3	96.4	86.3	89.1	84.6	85.2	85.1	87.8	85.0	85.7
**Frequency**	10 Hz											
**tan *d* (-)**	0.19	0.20	0.11	0.21	0.23	0.22	0.23	0.22	0.32	0.18	0.31	0.28
**Tg (°C)**	98.8	99.1	100.6	108.7	94.2	96.7	93.8	93.7	93.5	97.4	94.1	94.9

## Data Availability

Not applicable.
